# Study of Anti Cancer Property of *Scrophularia striata* Extract on the Human Astrocytoma Cell Line (1321)

**Published:** 2010

**Authors:** Abdulreza Ardeshiry lajimi, Mostafa Rezaie-Tavirani, Seyed Alireza Mortazavi, Mansoureh Barzegar, Seyed Hasan Moghadamnia, Mohamad Bagher Rezaee

**Affiliations:** a *Proteomics Research Center (PRC), Faculty of Paramedical Sciences, Shahid Beheshti University of Medical Sciences, Tehran, Iran.*; b * Department of Pharmaceutics, School of Pharmacy, Shahid Beheshti University of Medical Sciences, Tehran, Iran.*; c * Department of Cell and Molecular Biology, Khatam Universiy, Tehran, Iran.*; d *Institute of Forest and Rangelands, Tehran, Iran.*

**Keywords:** Astrocytoma, 1321 cell line, *Scrophularia striata *extract, Flow cytometry, Anticancer effect

## Abstract

There are considerable efforts to identify naturally occurring substances as new drugs in cancer therapy. Many components of medicinal plants have been identified that possess substantial anticancerous properties. This prompted us to investigate the effect of *Scrophularia striata *(an Iranian species belonging to the *Scrophulariace *family) extract on the growth of astrocyte cancer cell line (1321). The 1321 cell line were seeded in 96-well culture plates in the presence and absence of various concentrations of either leaf and seed filtered and unfiltered extract of *Scrophularia striata *to determine their probable anticancer effects in comparison with etoposide (chemical anticancer reagent). filtered leaf extract of *S. Striata *showed strong anticancer effect on 1321cell line as compared to control group (cells not exposed to extracts), and even the group (adenocarcinoma gastric cell line) exposed to etoposide. Unlike the leaf extract, the seed extract activated cell proliferation in all experiments. Flow cytometry findings indicated that apoptosis is the mechanism by which the leaf extract inhibits cell proliferation. Our findings indicate that both leaves and seeds of *S. Striata *contain both anti cancer and cell growth enhancing agents.

## Introduction

Cancer is the third leading cause of death worldwide, only preceded by cardiovascular disease, infectious and parasitic disease (1, 2). Cancer development processes are dependent on alteration in molecular, biochemical and cellular controls, such as elaboration of proteolytic enzymes necessary for invasion and progression of the tumor. Importance of proteolytic enzymes in tumor invasion is expressed as zymogens which must be proteolytically processed for activation (3-5). Chemotherapy is the treatment of disease, especially cancer, using chemical substances. These chemicals are capable of destroy cancer cells, keeping them from growing and spreading, shrinking the size of a tumor or relieving cancer symptoms. Chemotherapy can destroy or slow down the growth of normal cells, including cells of the hair, mouth, digestive system, as well as those of blood (6). Each person with cancer reacts differently to chemotherapy and its various side effects (7- 9). Fortunately, doctors now know many ways to reduce and even prevent these side effects. Oncologists are still looking for new anticancer drugs with more potent inhibitory and less side effects (10, 11). Presently, more than 50% of drugs come from one or several natural products of 25,000 plant species and 600 of them have anticancer properties. Natural products have been used by in traditional medicines as a source of remedies for thousands of years, dating back to ancient empires in Persia, Mesopotamia, Egypt, China, Greece, and Rome (12). These traditional medicinal preparation are made by boiling the plant material in water or soaking in alcohol (13, 14). One such preparation is a formula using a diterpene ester from Daphne macronata animal at investigating cytotoxic activity against lung and prostate cancer (15). The *Scrophulariaceae *is a large angiosperm family, which is widely distributed in deciduous and coniferous forests of central europe, central asia, and north america, especially in the mediterranean area, and is represented by about 3000 species and 220 genera (16). Some species of the family have been used since ancient times in traditional medicines to treat eczema, wounds, goiter, ulcers, cancer and fistulae. Some of them are boiled in milk to prepare a poultice which is applied to the abdomen to remove or reduce abdominal pain, whereas their aqueous extracts have been used as a bath to alleviate rheumatic pains. *Scrophulariaceae *species have been known to be rich in iridoid glycosides, mainly aucubin and catalpol (17). Iridoids represent a large group of cyclopentan-[c]-pyran monoterpenoids occurring as constituents of sympetalous plants including ornamental as well as wild ones. Their structures, properties and biosyntheses have been reviewed (18- 20). They have shown various biological activities such as antimicrobial, antitumoral, hemodynamic, choleretic, hepatoprotective and anti-inflammatory properties (21). There are promising reports of chemoprevention of skin and lung cancer by genipin, an iridoid obtained on hydrolysis of geniposide, a glycoside isolated from the fruits of Genipa americana and Gardenia jasmoindes (22, 23).

Prompted by these reports, we examined, the cytotoxic effect of *Scrophularia striata *on 1321 cell line in order to determine its probable anticancer properties. Furthermore, by studying the effect of this extract on fibroblast cell line, we tried to investigate its probable side effect. Our finding can be used for assessment of *Scrophularia striata *as a new anticancer drug.

## Experimental

*Materials*

The cell culture medium (DMEM), fetal bovine serum (FBS), penicillin and streptomycin were provided by Gibco BRL (Life Technologies, Paisley, Scotland). Cell lines were obtained from cell bank (Pastuer Institute,Tehran, Iran). 3-(4, 5-dimethyl-thiazol-2-yl)-2, 5-diphenyltetrazolium bromide (MTT), Annexin V-FLOUS staining kit (Cat. No. 11 988 549 001) was obtained from Roche Diagnostics GmbH (Germany).

*Plant material*

Aerial parts of *Scrophularia striata *were collected from Ilam province during the spring season. These aerial parts of *S. striata*, was exposed to sunlight, washed and put into plastic bags and immediately frozen at -20 °C. The plant material was then freeze-dried.

*Methods*

*Extraction of plant components*

The double distilled water (DW) was treated in a GFL system (1204, Germany). 8 mg and 10 mg of leaves and seeds were extracted with 80 mL and 100 mL of water at 65 °C for 1 h, respectively, then filtered through filter paper. The filtrate was then divided into two parts. One part was filtered through a 0.2 μm Milipore membrane filter, and the other one was kept unfiltered for cell cycle growth procedure.

*Cell culture*

The human astrocytoma cell line (1321) was cultured in the DMEM medium which had been treated with FBS (10%, v/v), streptomycin (100 μg/mL), penicillin (100 U/mL). The cells (5 × 10^3^) were seeded, in triplicate, into the 96 well plates and incubated at 37 °C under 5% CO2 atmosphere for 24 h. Then, the various concentrations (of leaves and seeds extract 0 as control (without the leaves or seed extract), 1, 3, 5, 7, 10 and 20 μg/mL), both of filtered and unfiltered (separated), were added to the cells a day and for incubation periods (24, 48 and 72 h). 

*Microscopic study*

In order to compare the cell morphology and pattern of cell distribution in the absence (without seed extract) and presence of the extract an inverted microscope (Ceti) was used. 

*Cell viability*

Cell viability was assessed by using a 3-(4, 5- dimethyl-thiazol-2-yl)-2, 5-diphenyltetrazolium bromide (MTT)-based colorimetric assay. Cells in 96-well plates (5000 cells/well) were exposed to various concentrations of extract substance (0 as control, 1, 3, 5, 7, 10 and 20 μg/mL), then incubated at 37°C under 5% CO2 atmosphere for 3 h. The 30 μL MTT solution (5 mg/mL in phosphate buffered saline) was added and further incubated for 4 h at 37 °C. After aspirating the supernatant from the wells, 100 μL dimethyl sulfoxide (DMSO) were added to dissolve of formazan crystals. Finally, The absorbance of each well was observed at 570-630 nm using an ELISA plate reader. 

The percentage of cytotoxicity was calculated using to the following formula: 

$\mathrm{\%Cytotoxicity}=(\frac{1-\mathrm{mean absorbance of toxicant}-\mathrm{treated cells})\times 100}{\mathrm{Mean absorbance of negative control}})$

% Viability = 100 - % Cytotoxicity 

*Flow cytometery analysis*

For flow cytometry analysis, 1321 cells were cultured into 6-well plates at a density of 1 × 10^6^ cells in the presence and absence of the cytotoxic agents for 48 h. All floated and adherent cells were harvested and centrifuged at 200 ×g for 10 min. Cell pellet was washed with 1X phosphate buffer saline solution and centrifuged at 200 ×g for 10 min. The cell pellet was then resuspended in 100 μL of Annexin V/FLUOS labeling solution (predilute 20 μL Annexin V/FLUOS labeling reagent in 1 mL incubation buffer and add 20 μL propidium iodide solution), and incubated at 15-25 °C for 10-15 min. It was then employed to analyze the cell population analyzed by flow cytometer (Bio-Rad, USA). Using 10 μM concentration of unfiltered *scrophularia striata *leaf extract giving 50% cytotoxicity (LC_50_) was selected to evaluate of apoptosis. FL_1_ and FL_2_ channels were used for detection of Annexin V/ FLUOS labeling solution, respectively. In this experiment, the cells were aspirated by PBS, and then 1×10^6^, 1321 cells were used. The samples were read in a FACS flow cytometer (USA) using 488 nm excitation and a 515 nm bandpass filter for fluorescein detection and a filter > 600 nm for propidium iodide detection. Analyses were performed by the software supplied in the instrument. 

*Statistical analysi*s 

Results were expressed as mean ± SD. Mean difference among groups was calculated by one-way and two-way variance analysis and p < 0.05 was considered statistically significant. 

## Results and Discussion

**Figure 1:**
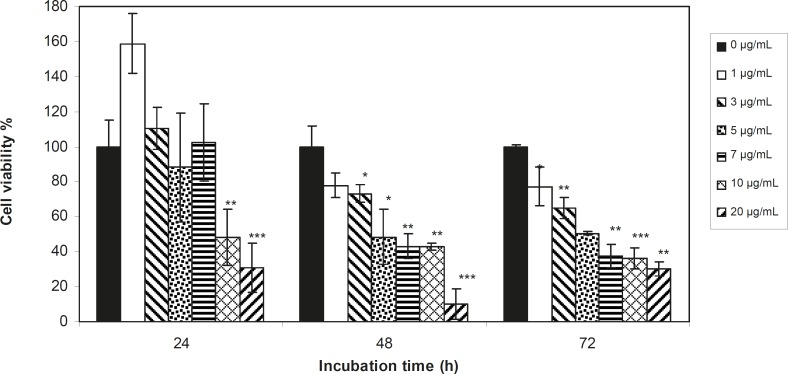
Viability percentage of 1321 Cell line in the presence of 0, 1, 3, 5, 7, 10 and 20 μg/mL concentrations of filtered leaf extract at 24, 48 and 72 h incubation times. Results are presented as mean ± SD. Significant levels are *p < 0.05; **p < 0.01 and ***p < 0.001

**Figure 2:**
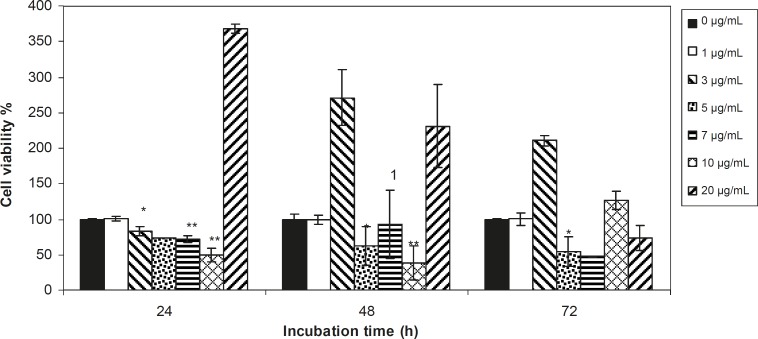
Viability percentage of 1321 Cell line in the presence of 0, 1, 3, 5, 7, 10 and 20 μg/mL concentrations of unfiltered leaf extract at 24, 48 and 72 h incubation times. Results are presented as mean ± SD. Significant levels are *p < 0.05; **p < 0.01 and ***p < 0.001

**Figure 3:**
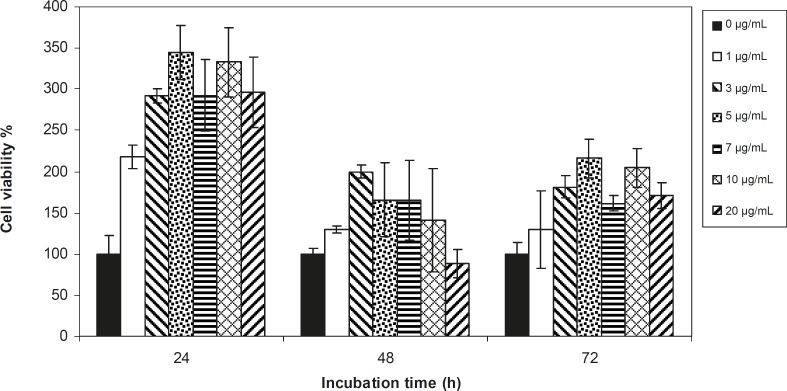
Viability percentage of 1321 Cell line in the presence of 0, 1, 3, 5, 7, 10 and 20 μg/mL concentrations of filtered seed extract at 24, 48 and 72 h incubation times. Results are presented as mean ± SD. Significant levels are *p < 0.05; **p < 0.01 and ***p < 0.001

**Figure 4:**
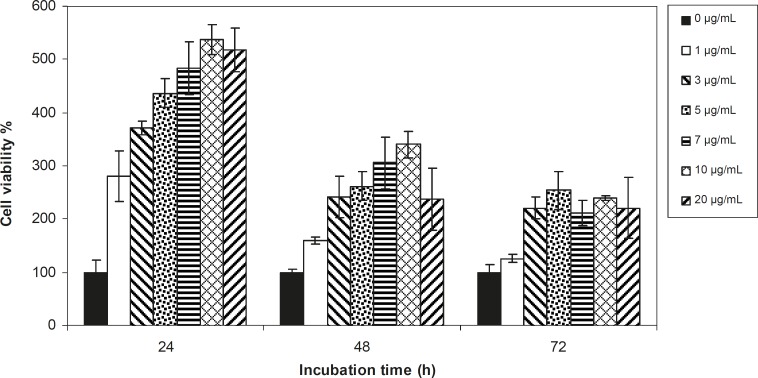
Viability percentage of 1321 Cell line in the presence of 0, 1, 3, 5, 7, 10 and 20 μg/mL concentrations of unfiltered seed extract at 24, 48 and 72 h incubation times. Results are presented as mean ± SD. Significant levels are *p < 0.05; **p < 0.01 and ***p < 0.001

Viability of 1321 cell line was studied in the presence of various concentrations of *S. Striata *extract at incubation times of 24, 48 and 72 h. Figures 1-2, represent viability of 1321 cell line in the presence of filtered and unfiltered leaf extract of *S. striata*, respectively. There are many evidences that correspond to the existence of differences between pharmacological properties of the leaves and seed of various plants (24-26). The experiment was repeated for seed extract as it was done for the leaf extract (see Figure 3-4). As shown in Figures 1-4, of all used the extracts used, only the filtered leaf extract had no regular cytotoxic effect on the 1321 cell line. LC_50_ values for the data in Figure 1 in comparison with the data from the effect of etoposide are tabulated in Table 1. 

**Table 1:** The LC_50_ values for the data at the Figure 1 in comparison with the data from the effect of etoposide are tabulated in this table

**LC**50	**24 h **	**48 h **
**Leaf **	6 μg/mL	9 μg/mL
**Etoposide **	75 μg/mL	40 μg/mL

**Figure 5:**
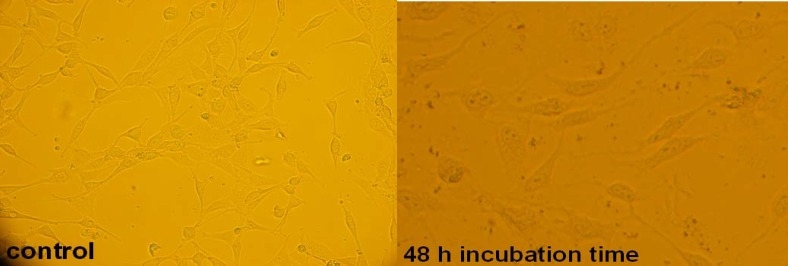
Microscopic view of 1321 cell line in the absence and presence of LC_50_ concentration of filtered leaf extract at 48 h incubation time

**Figure 6:**
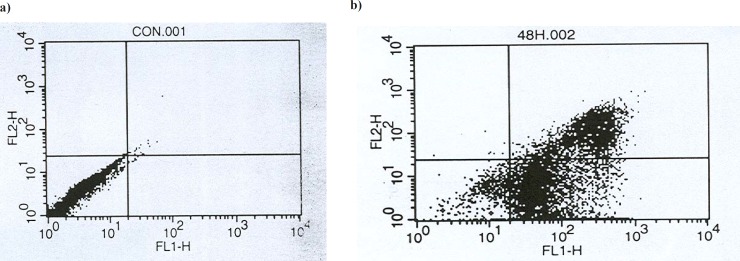
A flow cytometry scheme in evaluation of: (**a) **Control group (the cells in the absence of extract) (**b) **Sample cells (the cells in the presence of LC_50_ concentration of filtered leaf extract) after incubation for 48 h. The cells were harvested, stained with Annexin V/FLUOS (FL-I) and propidium iodide (PI, FL-2) and analyzed by flow cytometry. Four populations are resolved. Living cells or Annexin- V/FLUOS (-) /PI (-) [LL] are seen in the lower left quadrant. Cells that are Annexin V/FLUOS (+)/PI (-) [LR] are apoptotic (lower right). The cell population with Annexin V/FLUOS (+)/PI (+) [UR] has been described as necrotic or advanced apoptotic (upper right) and Annexin V/FLUOS (-)/PI (+) [UL] may be bare nucle cells in late necrosis, or cellular debris (upper left).

**Figure 7:**
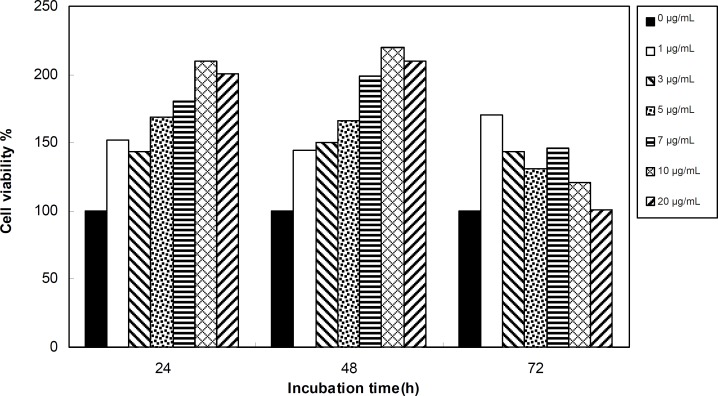
Viability percentage of Fibroblast cell line in the presence of 0, 1, 3, 5, 7, 10 and 20 μg/mL concentrations of filtered leaf extract at 24, 48 and 72 h incubation times. Results are presented as mean ± SD. Significant levels are *p < 0.05; **p < 0.01 and ***p < 0.001

There are many references that point to the effect of cytotoxic agents on the cell morphology and proliferation pattern (28- 30); so, the cytotoxicity effect of 5.5 μg/mL concentration (LC_50_ concentration) of filtered leaf extract was studied morphologically by microscopic method (see Figure 5), and cell death mechanism was studied by flow cytometric method (Figure 6). The amounts of cells that died by apoptosis and necrosis are tabulated in Table 2. To study the side effects of the filtered leaf extract on normal cells, the inhibitory property of 5.5 μg/mL concentration of extract was examined on human fibroblast cells (Figure 7). 

**Table 2:** Apoptotic index of the 1321 cancer cells treated with filtered leaf extract (9 μg/mL) for incubation time (48 h) in comparison with the control cells

**Time of incubation **	**Necrotic cells **	**Living cells **	**Apoptotic cells **
**48 h (control) **	0.002	0.989	0.009
**48 h **	27.56	6.41	65.93

An effective anticancer drug usually inhibits cell proliferation at a certain dose and also incubation times (31-32). As shown in Figures 1-4, only the filtered leaf extract of *S. striata *inhibited 1321 cell line proliferation. The seed extract had no cytotoxic effect, caused evokes cell proliferation (Figures 3, 4). These properties of leaf and seed extract of *S. striata *are similar to those of *S. deserti *(6, 33). The inhibitory effect of unfiltered leaf extract is accompanied by its stimulatory property (Figure 2). From Figure 2, it may be concluded that the stimulatory property of unfiltered leaf extract decreases with increasing incubation times. Yasunori *et al*. have reported that the presence of two substances with different effects in some parts of certain plants (34). Since the stimulatory effect of the leaf extract was eliminated by filtration, it can be concluded that the two different effects of the extract are attributable to two different substances. As the filter used for filtering the leaf extract was a 0.2 μm Milipore membrane filter, the inhibitor molecule must have a diameter less than 0.2 μm and the simulator agent a diameter greater than 0.2 μm. As shown in Figures 3 and 4, filtration had no effect on bringing about cell proliferation. Therefore, unlike the leaf extract, the stimulatory factor in seed is not similar to that in the leaf (the stimulatory factor in leaf was eliminated by filtration; but the factor in the seed was not). LC_50_ parameter is defined as the concentration of a chemical that attenuates cell survival to %50. It is a useful parameter for quantification of the drug effect on the cell survival (35). The LC_50_ values of filtered leaf extract on 1321 cell line are compared with the LC_50_ values of etoposide (36) and tabulated in Table 1. Their finding indicates that the extract is a potent anticancer reagent. For better understanding of the effect of filtered leaf extract on the 1321 cell line, the microscopic images of cells (Figure 5) are studied. As it is illustrated in Figure 5, the finding indicates that many cancer cells are dead and undergo granulation compared to the normal human fibroblast cell line. To achieve more complementary evidence, the flow cytometry experiment was performed. The 1321 cell line was treated with the filtered leaf extract consistent with LC_50_ value (9 μg/mL concentration of extract at 48 h incubation time). As shown in Figure 6, presence of extract induces apoptosis; so a major fraction of the cells that are Annexin V/FLOUS(+)/PI(-) [LR] are located in the lower right part of Figure 6 (b). Flow cytometry findings reveal that apoptosis is the main mechanism by which the extract brings about cell death. The calculated percentages of apoptotic and necrotic cells following treatment of 1321 cell line and control cells with unfiltered leaf extract are tabulated in Table 2 where it is shown that 66% of the cells undergo apoptosis in the presence of the extract. Since the magnitude of the side effect is a very important factor in chemotherapy (37), the cytotoxic effect of the filtered leaf extract on normal cells (human fibroblast cell) was assessed. Our finding (Figure 7) indicates that the extract does bring about fibroblast cell proliferation. 

In conclusion, it could be said that this finding demonstrated that *S. striata *extract affected cellular proliferation phenomena. In addition, leaf extract can inhibit 1321 cell line proliferation, so it can be used as an anti cancer drug with specific concentration. However, further investigations are to be needed to explore the molecular basis of this procedure. Besides, it is required to examine for other mechanisms, which are likely to be involved in growth inhibition of 1321 cells by *S. Striata***.**
